# Round the Hospitals

**Published:** 1917-08-04

**Authors:** 


### ROUND THE HOSPITALS.

Two notable instances of presentations made to nurses are reported this week. At an open-air Investiture at Aldershot His Majesty the King presented the Military Medal for bravery on the field to Nurse Annie Colhoun, who was the recipient of an outburst of sustained cheering, and was brought back, after receiving this high distinction, to be presented to Her Majesty Queen Mary. In France a posthumous honour has been bestowed by the French Army, in the shape of the Croix de Guerre with palm, upon Miss Mary Marshall, staff nurse, British Hospital No. 37, and Miss Margaret Dewar, a staff nurse at the same hospital. The French Army Orders describe Miss Marshall as a brave nurse who was the victim of her devotion to duty during an aerial bombardment of her hospital. When the enemy aeroplanes were signalled she went at once to the operation-room and made all the needful preparations. She continued her work during the intense bombardment, and was killed at her post. Nurse Dewar was mortally wounded during the bombardment of the hospital while protecting a patient's head with a pillow. Both these nurses' names were mentioned in General Milne's recent dispatch for distinguished service rendered during the past six months. They were members of Queen Alexandra's Imperial Military Nursing Service Reserve. Miss Marshall was trained at the Royal Southern Hospital, Liverpool, and Miss Dewar at the Western Infirmary, Glasgow. The Croix de Guerre has also been awarded to an American volunteer worker named Lovering, who was in charge of an American institution adjacent to the English hospital.

---

MISS AMY HUGHES, trained at St. Thomas's Hospital, has marked her nursing career by a commendable diffidence in reference to her own work and rewards. We place a very high value on this attribute, and have always done our best to respect Miss Hughes's wish in these matters. The reception of Miss Hughes by Queen Alexandra, and Her Majesty's presentation to her, on behalf of the Council of the Institute, of a purse in recognition of her services for twenty-two years in connection with the Queen Victoria's Jubilee Institute for Nurses, has an interest and importance to the British nursing world. The justification of this importance is testified to by the fact that the ladies and gentlemen of Her Majesty's household were in attendance when this high mark of recognition for personal service was made last week by Her Majesty Queen Alexandra, who is the very embodiment in practice of personal service, an attribute shared by His Majesty King George, as we have often demonstrated. We are glad to note that Viscount Goschen, the chairman, and Mrs. George Byron, the hon. secretary of the Institute; the Lady Northcote, president, and the Dowager Lady Dimsdale, vice-president of Queen Alexandra's Committee; the Countess of Kenmare, chairman of the Irish Advisory Committee; Lord Aberdare, representing the South Wales Nursing Association; Miss Bright, representing the Affiliated Associations in Lancashire and Cheshire; and Sir Dyce Duckworth, representing the Royal College of Physicians, were present at the ceremony and duly received by Her Majesty. Where was Scotland's representative? The practice of personal diffidence in regard to valuable service has indeed had a splendid recognition in the person of Miss Amy Hughes, who, we understand, will remain in office as General Superintendent of the Jubilee Institute until the end of September, being at present away on a well-earned holiday, somewhere, we hope, where the weather will be of the best and the surroundings everything calculated to minister health and happiness to herself. Miss Peterkin, Miss Hughes's successor, will enter upon her duties early in October.

"Shamus" in the *Weekly Irish Times*, has recently called attention to the demand for an active committee of Irish women to watch and regulate, and develop as far as possible, the better employment of women. Great hardship and low wages are at present the rule in Ireland, due mainly to the absence of facilities for training women workers for the occupations they wish to follow. Girl clerks, for instance, get a bad name owing to the absence of training, and the existing arrangement, whereby Irish hospitals find a large part of their support in the fees paid by probationers, who work for three years for little or nothing, makes distinctly for the degeneration of the nursing profession in Ireland. At present the probationers are entirely helpless; their parents do not wish to send them to London if they can avoid it, and when the Irish probationer ends her period of probation her salary is little, if any, better than that of a probationer in a London hospital. He condemns the various excuses which are made for the continuance of the present Irish system, and insists that the hours in Irish hospitals are not shorter than those in London, though the discipline in the former may be less. The present conditions result in the recruitment in Ireland of probationers of inferior type and educational attainment.

Nurses in Ireland are undoubtedly underpaid. Surgeon Wheeler, speaking to the City of Dublin Nursing Institution, declared that the rank and file of trained nurses were the hardest worked, worst paid, and, generally speaking, the least considered members of the community, and it was to be hoped that working nurses would take an active interest in the establishment of the Irish Nursing College on democratic lines. The whole nursing system in Ireland requires reorganisation and reconsideration from top to bottom, in the interests of the public, as well as of the Irish nursing profession.

The present urgent call for the registration of nurses was emphasised by a case before the Central Criminal Court last week. Louisa Davies and Grace Jenkins, both thirty-one, described as hospital nurses, who pleaded guilty to a charge of procuring a miscarriage in a married woman, Mrs. Armstrong, Jenkins's sister, were each sentenced to two months' imprisonment in the second division. Mr. Justice Lawrence said the prisoners seemed to have fallen into committing the offence by reason of their affection for one another, and for Mrs. Armstrong, the victim, who is dead. It does not appear where the prisoners were trained, nor if any and what steps will be taken, in the absence of a central authority, to make it impossible for these women, on their discharge, to continue to describe themselves as hospital nurses and to follow their calling.

It is a notable and historical fact that Mrs. Harley, who was killed by an enemy shell at Monastir last March, has had a monument erected to her memory in acknowledgment of her virtues and of her unforgettable services to the Serbian people. The memorial is a solid piece of masonry, consisting of blocks of grey granite cemented together, and surmounted by a white marble cross. Two marble bases bear the following inscription in gold letters, in the Serbian and English languages:—"To the victim of barbarians—a generous lady, a great benefactor of the Serbian people, and a great lady. On your tomb, instead of flowers, the gratitude of the Serbs shall blossom. For your wonderful deeds your name shall be known from generation to generation.—From the Officers at the Serbian Base." A large congregation attended an impressive full choral Requiem celebrated by the Serbian clergy over the grave of Mrs. Harley. Amongst those present were officers of the Serbian Army, British and French medical officers, several British chaplains, a goodly number of British, French, and Serbian Red Cross sisters, including Mrs. Harley's daughters, together with hospital attendants, numerous Serbians, men and women, and the Royal Serbian Guard. "She did what she could."

### The "Choker" Collar.

*To the Editor of* The Hospital.

Sir,—Seeing in this week's Hospital an account of the "choker" collar by a Red Cross matron, and as she asks for expressions on the subject I offer mine. I am a trained nurse of twenty-one years' experience, and during my training, various posts held since, and in private nursing, I have never experienced any inconvenience from a starched collar; neither have I heard any first-class nurses complain, and I seldom come in contact with second-rate nurses. I have not seen the choker of the modern nurse, and I have never known probationers to be allowed to unfasten their collars when on duty, and if I had unfastened mine during my training the sisters would have said I was slovenly. With the low-necked blouses that are worn now, I can quite imagine those just entering the nursing profession would find a collar a little uncomfortable at first, but they would soon get used to it. I think some of the uniforms now worn are more of a fancy dress than a uniform, and I should be very sorry to see myself in such a costume, for they are anything but smart. My collars are two inches deep, or a fraction under. I, too, will be interested to hear what others have to say on the subject, especially the matrons of our large hospitals and infirmaries.—Yours faithfully,

C. F. G. G. F.

*** It is our invariable practice to pay for all items of news which we may publish. The minimum payment is 5s. Paragraphs of about twenty lines in length—that is to say, of 150 words or less—are preferred. In this section during the war we propose to give 10s. to the correspondent who may send us in any week the best incident connected with hospital life and work. In this way we hope to afford to all practical aid and encouragement; while those who reciprocate will be able to render us invaluable service. Contributions should be addressed to The Editor, The Hospital, 28 and 29 Southampton Street, Strand, London, W.C., and, to be in time for the current week's issue, should reach him by the first post on Mondays.

